# Cardiac magnetic resonance imaging in repaired tetralogy of Fallot: A longitudinal midterm follow-up study

**DOI:** 10.1371/journal.pone.0308362

**Published:** 2024-12-19

**Authors:** Golnaz Houshmand, Rahem Rahmati, Elahe Meftah, Fatemeh Zarimeidani, Mohammadrafi Khorgami, Seyyed Mojtaba Ghorashi, Amir Fazeli, Negar Omidi, Sedigheh Saedi, Marzieh Motevalli, Majid Maleki, Hamidreza Pouraliakbar

**Affiliations:** 1 Rajaie Cardiovascular Medical and Research Center, Iran University of Medical Sciences, Tehran, Iran; 2 Students Research Committee, Shahrekord University of Medical Sciences, Shahrekord, Iran; 3 Cardiovascular Disease Research Institute, Tehran Heart Center, Tehran University of Medical Science, Tehran, Iran; 4 Students’ Scientific Research Center, Tehran University of Medical Sciences, Tehran, Iran; 5 Cardiovascular Disease Research Institute, Cardiac Primary Prevention Research Center, Tehran University of Medical Science, Tehran, Iran; All India Institute of Medical Sciences, INDIA

## Abstract

**Background:**

Biventricular dysfunction is frequent in patients with repaired tetralogy of Fallot, necessitating routine imaging to monitor for worsening conditions and determine whether procedures like pulmonary valve replacement (PVR) are needed. This study aimed to highlight the parameters of cardiac magnetic resonance imaging (CMR) and their association with adverse outcomes in the midterm follow-up of these patients.

**Methods:**

This longitudinal study recruited all patients with a history of tetralogy of Fallot total correction (TFTC) who had two CMR images at a minimum three-month interval at Rajaie Center from 2007 through 2017.

**Results:**

Fifty-six patients at a mean age of 15.23 ± 11.66 years at TFTC and a 1:1 gender distribution were assessed. Regarding adverse events, PVR was done on 18 patients (32%). Right and left ventricular dysfunction occurred in 43 (76.8%) and 18 (32.1%) patients, respectively. Death did not occur in the present study. RVOT fibrosis was present in 47 (92.2%). The stroke volume of both ventricles increased during the follow-up (*P* <0.05), although end-diastolic volume indices, end-systolic volume indices, ejection fractions, strain parameters, and ventriculoarterial coupling did not change significantly. Patients requiring PVR had a significantly higher end-systolic volume index in both ventricles (*P* <0.05) and a lower right-sided ejection fraction (*P* <0.01) and coupling ratio (*P* <0.05). The ejection fraction in the left ventricle correlated with global circumferential strain (*P* <0.01), while in the right ventricle, it correlated with global longitudinal strain (*P* <0.05) and the right-sided coupling ratio (*P* <0.01).

**Conclusion:**

Myocardial strain and ventriculoarterial coupling parameters could underscore personalized-approached therapy and follow-up to improve outcomes.

## Introduction

Tetralogy of Fallot (ToF) is the most common cyanotic congenital cardiac disease, affecting around 5 of 10 000 live newborns [1,2]. Tetralogy of Fallot total correction (TFTC) comprises ventricular septal defect closure and right ventricular (RV) muscle shave with or without patch enlargement to relieve the obstruction of the right ventricular outflow tract (RVOT). Postoperative complications include chronic volume overload, RVOT obstruction, ongoing pulmonary regurgitation and stenosis, RV extension, and tricuspid regurgitation. A previous long-term follow-up study also identified aortic root dilatation, left ventricular (LV) dysfunction, and arrhythmias [3].

Myocardial wall motion analysis is crucial in determining ventricular contractile activity [4]. Myocardial strain is defined as the ratio of length change from the resting state to the contracting state of the cardiac muscle [5] In contrast to ejection fraction (EF), measuring global longitudinal strain (GLS), global circumferential strain (GCS), or global radial strain (GRS) directions allows for a more comprehensive and detailed examination of the spatial components of contractility [5]. Quantifying myocardial deformation has provided additional insight into cardiac function in a range of subclinical cardiac diseases [4].

Time is of the essence for pulmonary valve replacement (PVR) to prevent RV and LV dysfunction [3,6]. RV function and geometry assessments are somehow challenging, with atrioventricular, ventriculoatrial, and interventricular interactions thus far suggested for predicting RV dysfunction [6]. Nevertheless, the mechanism and predictors of RV remodeling in TFTC remain poorly understood. Reduced GLS and GCS strain values of both LV and RV in repaired ToF were associated with adverse events [7].

Ventriculoarterial coupling measures the ventricle’s response to its afterload [8]. In patients with pulmonary hypertension, the ratio of stroke volume(SV) to end systolic volume(ESV) has been reported to be a more sensitive volume measure of ventriculoarterial coupling ratio than ventricular EF to detect alterations in function at the earliest stages of the disease. It also has been found as a prognostic ratio [9,10].

Drawing upon cardiac magnetic resonance imaging (CMR) indices, in the present study, we sought to highlight the association between CMR indices and the midterm outcomes of patients with TFTC. We also aimed to assess changes in the follow-up parameters and compare the differences between patients with PVR and those without it.

## Methods

The present longitudinal study identified and recruited all patients with a history of TFTC referred to Rajaie Cardiovascular Medical and Research Center for CMR from March 21, 2007, through March 21, 2017. The inclusion criteria were a history of TFTC with high-quality cine images and at least two CMR studies conducted at the aforementioned center. Based on previous studies, patients with an interval of less than a year between ToF repair and CMR or an interval of less than three months between the two CMR studies were excluded. The baseline characteristics of the patients, changes in their CMR induces, and adverse events during the postoperative follow-up period were recorded. The primary outcomes were PVR, sustained ventricular tachyarrhythmia requiring cardiovascular implantable electronic devices and death. The secondary outcomes were changes in RV, LV systolic function and volumes, pulmonary regurgitation. The ventricular end-diastolic volume index (EDVI) and EF changes were assessed in a subgroup of patients with PVR and compared with those without PVR.

### CMR acquisition

Cine images were used to evaluate ventricular volumetric studies. The images were produced by a Siemens MAGNETOM Avanto 1.5T (Siemens Healthcare Sector, Erlangen, Germany). As previously confirmed, balanced steady-state free-precession sequences in a short-axis stack were utilized to conduct functional imaging. Slice thicknesses of 6–8 mm, matrix sizes of 256 × 192, and minimal echo and repetition times were the typical imaging characteristics. Non-breath-hold phase-velocity mapping is used to quantify pulmonary regurgitation in a plane that transects the reconstructed pulmonary trunk. The Late Gadolinium Enhancement (LGE) was assessed 10 minutes after administering an intravenous injection of 0.15 mmol/kg Gadoterate meglumine (gadolinium-DOTA, Dotarem, Guerbet S.A., Paris, France) with inversion-recovery gradient echo sequence in short-axis stack, three long-axis view and right ventricular outflow (RVOT) images using magnitude and phase-sensitive inversion recovery reconstructions.

### CMR analysis

A commercially available workstation was employed to analyze the CMR images (Extended MR WorkSpace; the CVI42 software). The images were reviewed by a cardiologist specialized in CMR with five years of experience. The manual contouring of the endocardial boundaries at end-diastole and end-systole was used to determine LV and RV volumes and EF, respectively. The velocity flow mapping imaging of the main pulmonary artery was utilized to determine regurgitant volumes and fractions.The right ventriculoarterial coupling ratio was determined by dividing the RV stroke volume (SV) by the RV end-systolic volume index (RVESVI) [8–10]. LGE was considered present if there was enhancement in cross-cut or phase swap images. The RV/LV insertion points were considered nonspecific [11].

The actual body surface area was determined for individuals of varying ages and within healthy weight ranges using the Haycock formula.

### Strain analysis

The strain analysis of the LV was performed by loading the cine short axis, identifying the end-diastolic and end-systolic phases, and starting the tracking of the endocardial and epicardial boundaries. Cine two, three, and four-chamber views were loaded to perform longitudinal feature tracking, and the endocardial and epicardial boundaries were defined. The RV endocardial boundaries were tracked in the short-axis cine to determine RV circumferential strain, and the RV 4-chamber view and the attachment point of the tricuspid valve to the free and septal walls were tracked to determine RV longitudinal strain [12].

### Statistical analysis

Descriptive statistics were used to report categorical variables, while mean and standard deviation (SD) were utilized to report continuous variables. As Shapiro-Wilk test results rejected the normality of the sample, the correlation between the continuous variables in each follow-up was assessed using the Spearman rho. A comparison between patients with and without PVR was made using the Mann-Whitney *U* test. The categorical variables were compared between the patients with and without PVR using Fischer’s Exact test. For the comparison of changes between the two follow-ups, the McNemar and Wilcoxon signed-rank tests were employed for dichotomous and continuous variables, respectively. SPSS version 16 for Windows was used for the statistical analyses. The cutoff for significance was a *P* value of 0.05. Where available, an exact *P* value was calculated and reported.

### Ethics statement

Approval for the study protocol was obtained from the Research Ethics Committee of Rajaie Cardiovascular Medical and Research Center on 14.05.2022 (ethics code: IR.RHC.REC.1401.006). All participants provided written informed consent before inclusion, and the investigation conformed to the principles outlined in the Declaration of Helsinki. It is necessary to mention that we obtained written informed consent from parents or guardians in the case of children. Furthermore, all data were fully anonymized before being accessed After obtaining approval from the ethics committee, we gained access to the data for conducting the research project on 01.06.2022. It is essential to mention that as part of the initial health evaluation process at this center, we always inquire with the patients or their parents whether they consent to the anonymous use of their medical data in research projects.

## Results

Fifty-six patients with TFTC at a mean age of 15.23 ± 11.66 years (range: 1–41) were observed during the present study. The mean duration of the whole follow-up (the time between surgery and the last follow-up) and the mean duration between the two follow-ups (between the two CMRs) were 159.92 ± 109.34 months and 28.29 ± 17.66 months, respectively. The baseline characteristics and the prevalence of the outcomes in the studied patients are summarized in Table 1.

**Table 1:** The baseline characteristics and outcomes in the studied patients.

Variable	(N = 56)
**Male, No. (%)**	28 (50)
**Age at TFTC (y), mean (SD)**	15.23 (11.66)
Body surface area (m^2^), mean (SD)	1.38 (.39)
**Heart rate, mean (SD)**	82.21 (15.97)
**Weight (kg), mean (SD)**	46.40 (19.38)
**Height (cm), mean (SD)**	149.37 (21.13)
**Duration between the 2 follow-ups (mon), mean (SD)**	28.29 (17.66)
**Time between surgery and the last follow-up (mon), mean (SD)**	159.92 (109.34)
**Right-sided aortic arch, No. (%)**	11 (19.6)
**PVR, No.(%)**	18 (32)
** Metallic valve**	5 (28)
** Biologic valve**	13 (72)
**Arrhythmia, No. (%)**	1 (2)
**Permanent pacemaker, No. (%)**	2 (3.6)
**Outcomes in the first follow-up, No. (%)**	
** RV failure**	49 (87.5)
** LV failure**	11 (19.6)
** Mild PR**	3 (5.8)
** Moderate PR**	11 (21.2)
** Severe PR**	38 (73.1)
** RVOT fibrosis**	47 (92.2)
** RVOT aneurysm**	22 (39.3)
**Outcomes in the second follow-up, No. (%)**	
** RV failure**	49 (87.5)
** LV failure**	18 (32.1)
** Mild PR**	3 (6.4)
** Moderate PR**	12 (25.5)
** Severe PR**	32 (68.1)
** RVOT aneurysm**	14 (25)

PVR, Pulmonary valve replacement; RV, Right ventricle; LV, Left ventricle; PR, Pulmonary regurgitation; RVOT, Right ventricular outflow tract.

Regarding the outcomes, one patient (2%) experienced ventricular tachyarrhythmia, and two (3.6%) received a permanent pacemaker. PVR was performed on 18 patients (32%), of whom 13 (72%) received a metallic valve, and 5 (28%) received a biological valve. The mean duration from TFTC to PVR was 12.66 ± 7.58 years, and the mean age at PVR was 15.68 ± 9.01 years. While assessing the outcomes in the CMRs, we detected reduced RVEF lower than 50% in 49 patients (87.5%) in both follow-ups. However, the prevalence of LV failure rose from 11 (19.6%) to 18 (32.1%) in the second follow-up. Mortality did not occur in any patient until the telephone follow-up. No patient showed LV myocardial fibrosis.

The characteristics of the patients with and without PVR are compared in Table 2. This comparison demonstrated a significantly higher RVESVI (*P* <0.05 in both follow-ups), RVEDVI (*P* <0.05), and pulmonary pressure gradient (*P* <0.01) in the patients with PVR than in those without PVR. Compared with the patients without PVR, those with PVR had a notably lower RVEF (*P* <0.01), right-sided coupling ratio (*P* <0.05), and weight (*P* <0.05). Other differences between the two groups were not statistically significant. The incidence of tachyarrhythmia and permanent pacemaker insertion was too small to perform any subgroup analysis.

**Table 2:** Comparisons of the indices in the patients with and without PVR.

Variable	Mean (SD) in PVR−	Mean (SD) in PVR+	Mann-Whitney *U*	*P* value
**RVEDVI**	123.92 (28.26)	145.28 (44.11)	220.5	.032*
**RVESVI**	63.08 (21.62)	84.72 (38.73)	208.5	.018*
**RVEF**	47.87 (5.67)	43.11 (5.12)	188	.006**
**RVSV**	75.81 (32.91)	79.50 (31.59)	205.5	.667
**RV GLS**	-20.20 (5.91)	-18.50 (3.55)	61	.569
**RV GCS**	-15.40 (4.59)	-15.22 (2.81)	69	.910
**RV GRS**	44.53 (17.77)	33.46 (13.83)	45	.077
**PR RF**	34.91 (12.66)	38.30 (12.25)	186.500	.307
**PR Volume**	28.22 (13.30)	35.59 (8.75)	72.000	.062
**PUL PG**	13.11 (19.04)	24.15 (24.04)	7.500	.005**
**RV SV/ESVI**	1.59 (1.98)	0.97 (.33)	151	.035*
**BSA**	1.46 (.37)	1.23 (.38)	191	.057
**Age**	15.94 (11.92)	13.72 (11.27)	299.5	.461

PVR, Pulmonary valve replacement; RV, Right ventricle; EDVI, End-diastolic volume index; ESVI, End-systolic volume index; EF, Ejection fraction; GLS, Global longitudinal strain; GCS, Global circumferential strain; GRS, Global radial strain; SV, Stroke volume; BSA, Body surface area.

PVR+: Patients with PVR. PVR−: Patients without PVR.

*: Significant at *P* < .05.

**: Significant at *P* < .01.

A comparison between the two CMRs demonstrated a considerable increase in RV and LVSV in the second follow-up (*P* <0.01 and *P* <0.05, respectively).

The correlations between the variables of function, strain, and coupling ratio are summarized in Table 3. LVEF was significantly correlated with LVGCS (*P* <0.01 in both follow-ups), LVGRS (*P* <0.05 in the first follow-up), and LVGLS (*P* <0.01 in the second follow-up and *P* = 0.052 in the first follow-up). LVSV was negatively correlated with LVGLS and LVGCS in the second follow-up (*P* <0.05), although the mentioned correlation was not observed in the first follow-up. RVEF and RVSV negatively correlated with RVGLS in the second follow-up (*P* <0.05). The right-sided coupling ratio correlated with RVEF (*P* <0.01) and RVGRS (*P* <0.05) in the first follow-up and with RVGCS (*P* <0.05) in the second follow-up. RVOT fibrosis did not correlate with any of the variables of function, strain, or coupling ratio.

**Table 3:** Correlations between the variables of function, strain, and ventriculoarterial coupling.

Correlations in the First Follow-up		Correlation Coefficient	*P* value	Correlations in the Second Follow-up		Correlation Coefficient	*P* value
**LVEF**	LV GLS	-.401	.052	**LVEF**	LV GLS	-.549**	.004**
	LV GCS	-.560**	.00**		LV GCS	-.576**	.003**
	LV GRS	.461*	.023*		LV GRS	.327	.111
**LVSV**	LV GLS	-.405	.069	**LVSV**	LV GLS	-.415*	.044*
	LV GCS	-.308	.174		LV GCS	-.471*	.020*
	LV GRS	.162	.483		LV GRS	.396	.056
**RVEF**	RV GLS	.232	.276	**RVEF**	RV GLS	-.420*	.037*
	RV GCS	-.045	.835		RV GCS	-.348	.088
	RV GRS	.045	.835		RV GRS	.076	.720
	PR RF	-.211	.137		PR RF	.243	.100
	PR Volume	-.054	.765		PR Volume	.170	.289
	PUL PG	.407	.149		PUL PG	-.055	.828
	RV SV/ESVI	.525**	.000**		RV SV/ESVI	.196	.191
**RVSV**	RV GLS	.039	.870	**RVSV**	RV GLS	-.515*	.010*
	RV GCS	.168	.478		RV GCS	-.260	.220
	RV GRS	.175	.460		RV GRS	.233	.274
	PR RF	.009	.955		PR RF	.241	.111
	PR Volume	.301	.127		PR Volume	.558**	.000**
	PUL PG	.470	.105		PUL PG	-.108	.679
**RV SV/ESVI**	RV GLS	-.030	.891	**RV SV/ESVI**	RV GLS	-.389	.090
	RV GCS	-.199	.363		RV GCS	.448*	.048*
	RV GRS	.419*	.047*		RV GRS	.346	.135

LV, Left ventricle; EF, Ejection fraction; RV, Right ventricle; SV, Stroke volume; ESVI, End-systolic volume index; PR, pulmonary regurgitation; RF, regurgitation fraction; PUL PG, pulmonary pressure gradient.

*: Significant at *P* <0.05.

**: Significant at *P* <0.01.

## Discussion

Our longitudinal study found a meaningful correlation between LV and RV volumes and the global strain values of two CMR evaluations. LVGCS was correlated to LVEF, RVEF correlated with RV coupling and RVGLS, and RV coupling correlated with RVGRS and RVGCS. Consistent with an earlier study [13], patients who had increased RVESVI and RVEDVI were more likely to require PVR.

GCS, GLS, and GRS have been employed before to evaluate the function of the ventricles [6,14]. Still, their predictive value in repaired ToF remains uncertain [15]. There is also disagreement regarding the relationship between EF and strain patterns [6]. However, the strain could indicate myocardial dysfunction even with a normal EF [16]. Our study confirmed the correlation between all LV strain parameters and LVEF. LVGCS is the most relevant parameter to LVEF, as the ejection of a sizable amount of LVSV results from torsional and circumferential shape alterations [17]. Contrary to the other strain parameters, the correlation between LVEF and LVGCS was present in both CMRs. Thus, using LVGCS over LVEF in early myocardial dysfunction detection seems preferable.

While a prior investigation found GCS the most reproducible strain measurement on CMR feature tracking [18], we support the findings of Menting et al. [19] and Therrien et al. [20] in so far as they contended that GLS was a significant reflector of the overall function of the RV inflow and apical parts. In contrast to Orwat et al. [21] and Kalaitzidis et al. [22], we found no correlation between RVGCS and RVEF. Possible explanations for this observation include that, under normal conditions, the RV ejects blood generally by longitudinal shortening and that, under increasing RV pressure loading, the RV ejects blood primarily through circumferential contraction [23]. Similar to the findings of Orwat et al. [21], we confirmed that strain parameters offered more information than EF regarding their use in the early detection and prediction of dysfunction and adverse outcomes in patients with repaired ToF.

The parameters of RV function measured by CMR or echocardiography are load-sensitive, meaning they cannot tell us whether the RV is functioning effectively in response to a particular afterload. A higher RV afterload should result in greater contractility to preserve function and coupling [24]. Small ventricular function changes may be more easily detected with the coupling ratio (= SV/ESV) and may be superior to EF for quantifying RV contractile reserve [25]. Therefore, based on the approach of Sanz et al. [26] and supporting studies [25,27], we performed a noninvasive assessment of RV function using the ventriculoarterial coupling ratio. Patients with relatively preserved RV volumes and EFs may develop symptoms, which explains why identifying those with uncoupling may show a group that might profit from therapies before the onset of symptoms or borderline RV dilation and impairment [24].

CMR studies of volume and EF are considered the standard method for determining the risk level and optimal timing of PVR for individuals with chronic PR. The prognostic value of CMR has been extensively studied in the TFTC population. Nevertheless, CMR-based indices solely incorporate the preload with RVEDV and a load-dependent measure of systolic function as EF. These measurements do not consider the impact of abnormal RV afterload, which is present in TFTC patients [28]. Additionally, most TFTC patients exhibit normal or near-normal RV systolic function, signifying that RV systolic dysfunction is a delayed manifestation in the pathophysiological progression of TFTC-associated PR [29]. This statement suggests that a normal RV systolic function may not adequately reveal the RV’s adaptation to a given afterload [29]. In this regard, several studies found that the ventriculoarterial coupling ratio and myocardial strain indicate myocardial changes in TFTC patients despite having a normal or relatively preserved ventricular volumes and EF [16,21,24,25,28,29]. These markers suggest a significantly higher magnitude for coupling ratio and strain parameters.

In-depth, Orwat et al. [21] reported that combining CMR assessments of LV GCS and RV GLS identified a subgroup of TFTC patients with a significantly higher risk of adverse outcomes. They also found that RV GCS was significantly associated with symptomatic deterioration of at least one NYHA class during the follow-up period. Besides, our study uncovered a significant association between RV strain and the ratio of the right ventriculoarterial coupling. Therefore, it seems that RV strain serves as an indicator of PVR. Overall, these findings imply that evaluating strain and ventriculoarterial coupling ratio in cases where the EF is normal could provide valuable insights to make well-informed choices regarding the timely PVR.

Buddhe et al. [30] found a significant correlation between RV coupling and RVEDVI, ESVI, EF, and LVEF. In this regard, Panaioli et al. [29] demonstrated that RV coupling was worse in patients with repaired ToF requiring PVR despite normal RV systolic function. Panaioli and colleagues also concluded that before the manifestation of clinical symptoms and RV systolic dysfunction, RV coupling might be a reliable indicator of a continual deleterious RV reaction to enduring volume overload in repaired ToF. Since our population was slightly larger than this study, further studies are needed to address the controversy.

Similar to the studies of Berganza et al. [15] and Kalaitzidis et al. [22], the present study recruited a young sample at a mean age of 15.23 ± 11.66 years. Although Bokma et al. [13] reported that patients with TFTC at a younger age were more prone to undergo PVR, our analysis did not support this finding. Since the rate of adverse events rises with patient age, the relationships between other variables may reach statistical significance in samples with older patients. Furthermore, even in young individuals with repaired ToF, most physiological parameters may still be generally normal alongside a higher capacity of RV remodeling, highlighting the need for more sensitive diagnostic measures to assess young patients [31,32].

### Strengths and limitations

The strengths of the present study were the relatively long duration of the postoperative follow-up and the use of precise paraclinical imaging at two different time points. This study had some limitations as well. Firstly, the sample recruited may represent a subset of individuals with less advanced diseases. Secondly, the current study is a mid-cohort with few outcomes. Given the paucity of events and the small number of patients, further multivariate regressions of additional research are needed to confirm and validate the utility of our results. Thirdly, lack of a normal distribution, probably due to the small sample size, restricted the evaluation and analysis.

## Conclusion

Our study found that, except for the SV of the ventricles, other CMR parameters may not change significantly at a mean follow-up duration of about 28 months. It also suggests that CMR derived strain and ventriculoarterial coupling parameters could be potential personalized-based indicators of PVR requirement in patients with repaired ToF. Further studies are warranted to confirm the findings of the present study.

## Supporting information

S1 File The main dataset used in the study. (PDF)
