# Supplementary material for: Cardiac magnetic resonance imaging in repaired tetralogy of Fallot: A longitudinal midterm follow-up study
Source: PLoS One. 2024 Dec 19;19(12):e0308362. doi: 10.1371/journal.pone.0308362 (PMC11658471; doi:10.1371/journal.pone.0308362)
Supplement: S1 File — (PDF) [file pone.0308362.s001.pdf]

Code	Weight	Height	BSA	Heart_Rat	Age_at_TF	LVEDVI_1	LVESVI_1	LVEF_1	LVSV_1
160609	45	155	1.39	80	33	53	23	56	42
162626	68	174	1.81	67	32	47	21	56	48
211626	45	156	1.40	72	40	83	52	37	#NULL!
226398	83	190	1.80	86	29	89	40	55	104
246672	68	164	1.72	56	34	73	36	51	#NULL!
248894	62	170	1.71	60	35	78	41	60	63
253881	72	165	1.70	72	29	73	27	62	83
264187	61	160	1.65	86	25	75	33	55	69
270533	57	167	1.62	76	23	65	27	58	75
437506	70	162	1.75	#NULL!	34	40	14	65	43
472093	30	140	1.08	63	14	68	30	56	33
567622	#NULL!	#NULL!	1.59	#NULL!	15	87	32	63	86
569182	52	156	1.50	60	13	67	25	62	#NULL!
626237	50	170	1.57	#NULL!	10	59	25	57	35
629439	37	154	1.26	98	12	73	37	53	53
631612	75	175	1.80	78	31	75	43	42	61
646338	26	152	1.05	87	10	48	27	54	21
665709	65	170	1.75	130	13	56	24	58	57
683700	46	158	1.42	109	12	#NULL!	#NULL!	60	#NULL!
708517	#NULL!	#NULL!	#NULL!	#NULL!	9	50	23	52	28
731089	52	158	1.51	70	12	95	43	55	79
764837	28	140	1.00	78	8	68	37	44	32
767378	53	168	1.56	59	8	121	70	42	#NULL!
838057	33	146	1.18	#NULL!	10	70	35	49	35
861187	24	127	0.91	89	8	64	29	55	27
861835	23	120	1.40	#NULL!	1	66	26	60	35
871518	#NULL!	#NULL!	#NULL!	#NULL!	8	60	30	50	31
875681	#NULL!	#NULL!	#NULL!	#NULL!	7	79	35	55	#NULL!
890673	60	170	1.69	#NULL!	10	45	16	65	#NULL!
906020	52	150	1.48	90	6	63	28	55	36
923751	43	142	1.29	#NULL!	9	59	25	57	31
930024	16	120	0.72	77	6	102	42	58	43
940585	57	153	1.57	67	9	66	33	46	40
951093	20	126	0.84	76	7	66	77	55	21
990618	25	126	0.85	116	9	85	40	53	38
1095158	#NULL!	#NULL!	#NULL!	#NULL!	41	67	27	59	85
1124752	71	168	1.70	80	12	46	21	55	43
1149552	30	124	1.03	87	11	72	36	49	37
1181911	18	111	0.74	89	3	75	38	49	22
1230638	50	145	1.07	#NULL!	3	78	29	60	44
1230858	34	130	1.11	98	7	61	27	56	29
1261381	48	152	1.42	104	13	82	37	55	29
1270845	26	120	0.93	89	6	90	44	53	32
1308758	16	110	0.70	80	5	78	33	58	30
1326153	85	178	1.80	79	35	71	35	51	77
1327225	#NULL!	#NULL!	#NULL!	#NULL!	38	52	25	52	45

1337135	20	116	0.80	95	7	64	20	63	#NULL!
1339057	57	160	1.60	67	13	61	27	56	55
1351075	21	103	0.78	89	5	112	52	53	#NULL!
1355998	60	160	1.62	#NULL!	3	69	35	48	44
1376738	34	#NULL!	#NULL!	#NULL!	4	64	30	53	24
1404543	37	147	1.24	#NULL!	8	72	37	48	28
1423517	66	163	1.72	#NULL!	34	63	27	57	62
1438327	20	115	0.80	87	7	63	30	51	25
1481180	65	163	1.70	#NULL!	2	70	32	53	66
1512080	64	170	1.74	78	25	57	27	52	52

RVEDVI_1	RVESVI_1	RVEF_1	RVSV_1	RVOTAneu	RVOTFibro	RV_GLS_1	RV_GCS_1	RV_GRS_1	LV_GLS_1
85	42	52	62	1	1	-11.90	-9.98	35.85	-15.95
128	74	42	99	1	0	#NULL!	#NULL!	#NULL!	#NULL!
71	38	46	#NULL!	0	#NULL!	-11.74	-12.35	18.94	-6.80
126	59	53	140	0	1	-16.48	-15.19	33.24	-15.79
131	62	52	#NULL!	1	1	#NULL!	#NULL!	#NULL!	#NULL!
126	69	43	96	1	1	-21.32	-12.95	20.33	-11.31
113	46	59	121	0	1	-13.47	-10.36	19.97	-10.78
130	75	42	91	0	1	-23.97	-15.65	47.42	-15.04
80	40	50	71	1	1	-22.27	-15.98	62.24	-18.00
100	43	55	55	0	1	#NULL!	#NULL!	#NULL!	#NULL!
226	139	40	152	1	1	-18.88	-13.61	33.91	-7.73
113	61	45	82	0	1	#NULL!	#NULL!	#NULL!	#NULL!
107	53	50	#NULL!	1	1	#NULL!	#NULL!	#NULL!	#NULL!
118	61	44	46	1	#NULL!	#NULL!	#NULL!	#NULL!	#NULL!
137	66	52	90	0	1	#NULL!	#NULL!	#NULL!	#NULL!
191	107	44	161	0	1	-28.20	-21.64	45.30	-9.46
128	59	50	69	1	1	-16.31	-16.60	28.21	-10.25
101	93	48	48	0	1	-17.95	-23.95	32.41	-10.26
77	43	44	#NULL!	0	1	-17.21	-21.29	35.50	-15.78
102	6	40	72	1	1	-13.45	-7.47	22.40	-10.16
139	57	58	123	0	1	-19.70	-16.07	39.00	-14.39
136	86	37	53	0	1	#NULL!	#NULL!	#NULL!	#NULL!
283	186	34	#NULL!	1	1	#NULL!	#NULL!	#NULL!	#NULL!
102	54	47	50	0	#NULL!	#NULL!	#NULL!	#NULL!	#NULL!
130	74	43	68	1	1	-21.14	-17.12	32.54	-8.85
144	66	53	67	1	0	#NULL!	#NULL!	#NULL!	#NULL!
146	84	42	62	0	1	#NULL!	#NULL!	#NULL!	#NULL!
209	128	40	#NULL!	1	1	#NULL!	#NULL!	#NULL!	#NULL!
101	40	60	#NULL!	0	1	#NULL!	#NULL!	#NULL!	#NULL!
87	35	45	44	0	1	#NULL!	#NULL!	#NULL!	#NULL!
133	67	49	61	1	#NULL!	#NULL!	#NULL!	#NULL!	#NULL!
235	156	34	58	0	1	-16.89	-14.45	25.80	-10.73
106	52	51	68	0	1	#NULL!	#NULL!	#NULL!	#NULL!
58	94	40	73	1	1	-20.22	-14.95	35.38	-10.60
125	72	43	45	0	1	-25.31	-19.30	53.88	-13.91
115	62	46	117	0	1	#NULL!	#NULL!	#NULL!	#NULL!
135	77	42	99	0	1	#NULL!	#NULL!	#NULL!	#NULL!
181	103	43	80	1	0	#NULL!	#NULL!	#NULL!	#NULL!
121	68	43	31	0	1	#NULL!	#NULL!	#NULL!	#NULL!
136	78	43	52	0	1	#NULL!	#NULL!	#NULL!	#NULL!
114	54	50	60	1	0	-24.87	-15.56	59.11	-13.60
130	72	44	#NULL!	1	1	-17.93	-13.38	30.56	-18.07
124	65	48	47	1	1	-14.53	-11.27	18.81	-8.50
144	71	50	46	0	#NULL!	#NULL!	#NULL!	#NULL!	#NULL!
115	66	41	101	0	1	-20.00	-14.50	36.57	-11.63
138	73	46	101	0	1	#NULL!	#NULL!	#NULL!	#NULL!

128	69	46	#NULL!	0	1	-21.25	-17.20	34.50	-17.70
129	54	58	120	0	1	#NULL!	#NULL!	#NULL!	#NULL!
120	59	51	#NULL!	0	1	#NULL!	#NULL!	#NULL!	#NULL!
130	62	52	89	0	1	#NULL!	#NULL!	#NULL!	#NULL!
116	73	37	29	0	1	#NULL!	#NULL!	#NULL!	#NULL!
134	74	44	48	0	1	-31.10	-16.80	18.20	-13.41
95	50	47	47	0	1	#NULL!	#NULL!	#NULL!	#NULL!
115	59	48	44	1	1	#NULL!	#NULL!	#NULL!	#NULL!
171	100	41	125	1	1	#NULL!	#NULL!	#NULL!	#NULL!
90	46	48	76	0	1	#NULL!	#NULL!	#NULL!	#NULL!

LV_GCS_1	LV_GRS_1	PR_RF_1	PR_volume	PUL_PG_1	Left_coupl	Right_coup	LVEF1_CA	RVEF1_CA	PR_CAT_1
-17.98	40.16	41.00	33.00	#NULL!	1.83	1.48	0	0	3
#NULL!	#NULL!	39.00	41.50	13.50	2.29	1.34	0	1	3
-9.74	25.89	20.00	#NULL!	#NULL!	#NULL!	#NULL!	1	1	2
-18.56	46.90	#NULL!	#NULL!	25.00	2.60	2.37	0	0	#NULL!
#NULL!	#NULL!	42.00	#NULL!	#NULL!	#NULL!	#NULL!	0	0	3
-12.19	33.45	30.00	#NULL!	#NULL!	1.54	1.39	0	1	2
-16.99	42.13	28.00	#NULL!	#NULL!	3.07	2.63	0	0	2
-17.50	40.72	32.00	28.00	5.00	2.09	1.21	0	1	3
-20.65	54.60	3.00	2.20	8.00	2.78	1.78	0	1	1
#NULL!	#NULL!	38.00	37.00	#NULL!	3.07	1.28	0	0	3
-10.98	27.12	52.00	44.00	#NULL!	1.10	1.09	0	1	3
#NULL!	#NULL!	38.00	28.00	13.00	2.69	1.34	0	1	3
#NULL!	#NULL!	30.00	38.00	#NULL!	#NULL!	#NULL!	0	1	2
#NULL!	#NULL!	43.00	32.00	#NULL!	1.40	0.75	0	1	3
#NULL!	#NULL!	42.00	46.00	#NULL!	1.43	1.36	0	0	3
-9.44	30.97	75.00	#NULL!	#NULL!	1.42	1.50	1	1	3
-16.33	43.76	45.00	45.00	9.00	0.78	1.17	0	1	3
-12.44	35.22	27.00	38.00	#NULL!	2.38	0.52	0	1	2
-21.29	68.60	15.00	16.00	#NULL!	#NULL!	#NULL!	0	1	1
-12.58	37.19	33.00	24.00	14.00	1.22	12.00	0	1	3
-15.23	47.00	34.00	#NULL!	#NULL!	1.84	2.16	0	0	3
#NULL!	#NULL!	50.00	36.00	3.00	0.86	0.62	1	1	3
#NULL!	#NULL!	30.00	33.00	#NULL!	#NULL!	#NULL!	1	1	2
#NULL!	#NULL!	45.00	24.47	#NULL!	1.00	0.93	1	1	3
-15.17	28.60	46.00	34.00	#NULL!	0.93	0.92	0	1	3
#NULL!	#NULL!	33.00	21.00	#NULL!	1.35	1.02	0	0	3
#NULL!	#NULL!	49.00	38.10	#NULL!	1.03	0.74	1	1	3
#NULL!	#NULL!	43.00	26.09	#NULL!	#NULL!	#NULL!	0	1	3
#NULL!	#NULL!	30.00	17.00	#NULL!	#NULL!	#NULL!	0	0	3
#NULL!	#NULL!	8.50	#NULL!	#NULL!	1.29	1.26	0	1	1
#NULL!	#NULL!	55.00	#NULL!	#NULL!	1.24	0.91	0	1	3
-16.49	41.18	#NULL!	#NULL!	#NULL!	1.02	0.37	0	1	3
#NULL!	#NULL!	30.00	#NULL!	#NULL!	1.21	1.31	1	0	2
-12.35	38.79	60.00	#NULL!	1.00	0.27	0.78	0	1	3
-13.38	41.30	42.00	33.70	#NULL!	0.95	0.63	0	1	3
#NULL!	#NULL!	26.70	21.00	21.00	3.15	1.89	0	1	3
#NULL!	#NULL!	42.00	36.70	#NULL!	2.05	1.29	0	1	3
#NULL!	#NULL!	43.67	38.00	8.00	1.03	0.78	1	1	3
#NULL!	#NULL!	21.50	12.00	12.00	0.58	0.46	1	1	2
#NULL!	#NULL!	40.00	#NULL!	#NULL!	1.52	0.67	0	1	3
-16.50	41.67	41.40	29.50	11.10	1.07	1.11	0	1	3
-19.79	47.39	25.00	19.00	11.00	0.78	#NULL!	0	1	2
-9.92	23.90	54.00	36.00	#NULL!	0.73	0.72	0	1	3
#NULL!	#NULL!	30.00	#NULL!	#NULL!	0.91	0.65	0	1	2
-13.49	23.66	40.00	#NULL!	#NULL!	2.20	1.53	0	1	3
#NULL!	#NULL!	33.00	#NULL!	#NULL!	1.80	1.38	0	1	3

-18.13	51.80	27.00	#NULL!	#NULL!	#NULL!	#NULL!	0	1	2
#NULL!	#NULL!	41.00	#NULL!	#NULL!	2.04	2.22	0	0	3
#NULL!	#NULL!	35.00	#NULL!	#NULL!	#NULL!	#NULL!	0	0	3
#NULL!	#NULL!	#NULL!	#NULL!	#NULL!	1.26	1.44	1	0	#NULL!
#NULL!	#NULL!	#NULL!	#NULL!	#NULL!	0.80	0.40	0	1	#NULL!
-11.60	41.42	45.00	27.00	#NULL!	0.76	0.65	1	1	3
#NULL!	#NULL!	16.00	10.00	#NULL!	2.30	0.94	0	1	2
#NULL!	#NULL!	52.00	#NULL!	#NULL!	0.83	0.75	0	1	3
#NULL!	#NULL!	#NULL!	#NULL!	#NULL!	2.06	1.25	0	1	#NULL!
#NULL!	#NULL!	34.00	67.00	#NULL!	1.93	1.65	0	1	3

PR_severit	LVEDVI_2	LVESVI_2	LVEF_2	LVSV_2	RVEDVI_2	RVESVI_2	RVEF_2	RVSV_2	RVOTAneu
3	53	21	62	48	115	56	56	84	1
3	59	24	59	64	106	49	54	103	0
2	90	66	26	33	72	40	44	44	0
#NULL!	92	46	50	95	131	76	42	112	1
3	73	35	52	38	134	64	50	69	0
2	110	55	50	95	109	60	45	84	0
2	75	35	54	#NULL!	117	60	40	#NULL!	1
3	70	33	54	62	132	78	41	90	1
1	74	28	62	75	82	35	57	77	0
3	46	22	53	41	85	42	50	70	0
3	68	34	49	36	198	119	39	84	1
3	51	24	53	42	122	69	44	84	0
2	64	32	50	50	105	56	47	76	0
3	78	37	53	60	164	99	39	95	0
3	81	39	52	58	137	61	55	104	0
3	62	36	40	50	177	103	41	145	1
3	39	24	37	22	126	69	45	85	0
2	52	24	53	53	99	52	47	88	0
1	43	17	60	39	62	35	43	49	0
3	58	33	43	45	118	81	32	66	0
3	79	33	62	79	131	56	55	121	0
3	73	35	52	41	128	67	47	#NULL!	0
2	71	40	43	48	131	105	19	40	0
3	50	20	59	34	107	44	58	73	0
3	70	30	57	45	155	71	54	95	0
3	59	25	57	35	134	41	47	65	0
3	56	23	57	43	138	77	44	80	0
3	84	41	51	40	222	97	37	77	0
3	65	22	66	47	102	32	69	77	1
1	78	38	50	39	90	53	41	37	0
3	63	28	55	41	139	72	48	78	1
3	98	45	54	52	186	124	33	60	0
3	58	34	43	39	82	39	51	66	0
3	77	45	41	26	239	152	36	72	0
3	66	32	51	36	116	61	48	59	0
3	70	31	56	84	144	79	45	140	0
3	51	28	45	41	136	67	50	126	0
3	64	32	49	42	144	86	40	76	0
2	72	32	56	40	106	64	40	42	1
3	110	42	61	67	145	85	41	60	1
3	57	23	58	49	92	46	49	65	1
2	78	25	68	77	116	50	56	94	1
3	77	35	55	40	192	102	46	84	0
2	73	31	57	29	145	73	50	50	1
3	75	38	49	76	125	75	39	101	0
3	57	28	51	46	146	75	48	110	2

2	73	29	61	46	162	89	45	75	0
3	70	30	57	40	126	50	58	75	0
3	144	64	55	63	160	68	57	72	0
#NULL!	56	30	46	36	114	53	53	87	0
#NULL!	54	22	60	#NULL!	106	62	41	#NULL!	0
3	82	42	48	38	153	86	44	64	0
2	51	22	56	48	88	56	36	58	0
3	78	29	62	42	153	79	48	64	0
#NULL!	70	32	53	66	171	100	41	125	0
3	66	32	50	60	109	60	45	87	1

RV_GLS_2	RV_GCS_2	RV_GRS_2	LV_GLS_2	LV_GCS_2	LV_GRS_2	PR_RF_2	PR_volume	PUL_PG_2	Left_coupl
-27.53	-13.79	63.45	-15.13	-16.92	46.30	39.00	33.00	#NULL!	2.29
#NULL!	#NULL!	#NULL!	#NULL!	#NULL!	#NULL!	41.50	38.60	9.90	2.67
-16.22	-8.90	24.20	-8.23	-11.86	22.49	#NULL!	#NULL!	#NULL!	0.50
-23.60	-10.87	48.15	-14.88	-16.99	46.28	25.00	33.00	#NULL!	2.07
#NULL!	#NULL!	#NULL!	#NULL!	#NULL!	#NULL!	34.00	43.00	12.00	1.09
-23.18	-11.87	21.16	-14.02	-14.60	47.29	14.00	13.00	#NULL!	1.73
-26.21	-7.09	86.73	-16.23	-14.96	42.28	26.00	27.00	5.00	#NULL!
-19.76	-14.57	32.74	-16.17	-18.14	37.31	37.00	40.00	16.00	1.88
#NULL!	#NULL!	#NULL!	#NULL!	#NULL!	#NULL!	#NULL!	#NULL!	#NULL!	2.68
#NULL!	#NULL!	#NULL!	#NULL!	#NULL!	#NULL!	42.00	41.00	#NULL!	1.86
-19.56	-14.74	33.00	-12.25	-13.99	31.79	40.00	54.00	#NULL!	1.06
#NULL!	#NULL!	#NULL!	#NULL!	#NULL!	#NULL!	26.00	23.00	10.00	1.75
#NULL!	#NULL!	#NULL!	#NULL!	#NULL!	#NULL!	#NULL!	#NULL!	#NULL!	1.56
#NULL!	#NULL!	#NULL!	#NULL!	#NULL!	#NULL!	#NULL!	#NULL!	#NULL!	1.62
-25.23	-21.46	51.46	-16.22	-16.94	44.00	37.00	57.00	73.00	1.49
-23.09	-26.07	57.84	-7.53	-10.60	31.00	80.00	#NULL!	8.00	1.39
-28.53	-17.80	67.45	-14.22	-16.57	38.21	34.00	22.00	#NULL!	0.92
-18.00	-20.90	29.31	-9.79	-16.12	40.53	33.00	39.08	#NULL!	2.21
-21.40	-14.35	42.00	-13.66	-19.24	5.38	#NULL!	16.00	16.00	2.29
-19.22	-10.00	31.36	-11.96	-12.90	31.93	30.00	17.00	#NULL!	1.36
-25.60	-15.65	25.25	-13.11	-16.47	37.13	32.00	36.00	#NULL!	2.39
#NULL!	#NULL!	#NULL!	#NULL!	#NULL!	#NULL!	50.00	#NULL!	#NULL!	1.17
-16.18	-11.28	26.53	-5.86	-13.29	23.10	24.70	21.00	#NULL!	1.20
#NULL!	#NULL!	#NULL!	#NULL!	#NULL!	#NULL!	43.00	#NULL!	#NULL!	1.70
-32.10	-16.50	29.00	-16.20	-18.00	42.20	39.00	40.00	#NULL!	1.50
#NULL!	#NULL!	#NULL!	#NULL!	#NULL!	#NULL!	35.00	41.00	#NULL!	1.40
#NULL!	#NULL!	#NULL!	#NULL!	#NULL!	#NULL!	41.00	37.70	5.59	1.87
#NULL!	#NULL!	#NULL!	#NULL!	#NULL!	#NULL!	36.00	21.00	9.00	0.98
#NULL!	#NULL!	#NULL!	#NULL!	#NULL!	#NULL!	43.00	26.00	8.00	2.14
#NULL!	#NULL!	#NULL!	#NULL!	#NULL!	#NULL!	10.00	3.00	#NULL!	1.03
#NULL!	#NULL!	#NULL!	#NULL!	#NULL!	#NULL!	50.00	21.62	#NULL!	1.46
-11.15	-8.70	14.53	-14.26	-18.73	49.90	48.00	#NULL!	#NULL!	1.16
#NULL!	#NULL!	#NULL!	#NULL!	#NULL!	#NULL!	36.00	21.00	2.00	1.15
-16.44	-20.22	32.22	-12.14	-10.81	38.62	65.00	48.00	#NULL!	0.58
-21.58	-19.91	40.91	-8.88	-11.36	32.91	25.00	15.00	9.00	1.13
#NULL!	#NULL!	#NULL!	#NULL!	#NULL!	#NULL!	35.00	37.98	#NULL!	2.71
#NULL!	#NULL!	#NULL!	#NULL!	#NULL!	#NULL!	34.50	33.80	#NULL!	1.46
#NULL!	#NULL!	#NULL!	#NULL!	#NULL!	#NULL!	41.00	#NULL!	#NULL!	1.31
#NULL!	#NULL!	#NULL!	#NULL!	#NULL!	#NULL!	22.00	6.36	#NULL!	1.25
#NULL!	#NULL!	#NULL!	#NULL!	#NULL!	#NULL!	35.00	25.00	#NULL!	1.60
-19.51	-16.57	36.03	-14.70	-14.43	31.06	#NULL!	#NULL!	#NULL!	2.13
-17.93	-13.38	30.56	-18.07	-19.79	47.39	16.00	9.30	7.00	3.08
-19.39	-15.77	24.99	-8.45	-11.40	32.47	46.00	36.50	15.00	1.14
#NULL!	#NULL!	#NULL!	#NULL!	#NULL!	#NULL!	30.00	15.00	15.00	0.94
-20.15	-13.89	36.14	-13.37	-14.96	28.96	26.00	28.00	#NULL!	2.00
#NULL!	#NULL!	#NULL!	#NULL!	#NULL!	#NULL!	56.00	44.00	#NULL!	1.64

-18.80	-18.00	35.70	-13.45	-14.00	35.70	30.00	#NULL!	#NULL!	1.59
#NULL!	#NULL!	#NULL!	#NULL!	#NULL!	#NULL!	42.00	#NULL!	#NULL!	1.33
#NULL!	#NULL!	#NULL!	#NULL!	#NULL!	#NULL!	42.00	20.00	#NULL!	0.98
#NULL!	#NULL!	#NULL!	#NULL!	#NULL!	#NULL!	#NULL!	#NULL!	#NULL!	1.20
#NULL!	#NULL!	#NULL!	#NULL!	#NULL!	#NULL!	#NULL!	#NULL!	#NULL!	#NULL!
-24.13	-23.06	48.60	-11.88	-13.90	39.80	35.00	18.22	73.00	0.90
#NULL!	#NULL!	#NULL!	#NULL!	#NULL!	#NULL!	15.00	12.60	#NULL!	2.18
#NULL!	#NULL!	#NULL!	#NULL!	#NULL!	#NULL!	29.60	20.50	8.76	1.45
#NULL!	#NULL!	#NULL!	#NULL!	#NULL!	#NULL!	#NULL!	#NULL!	#NULL!	2.06
#NULL!	#NULL!	#NULL!	#NULL!	#NULL!	#NULL!	37.00	33.00	#NULL!	1.88

Right_coup	PR_CAT_2	PR_severit	RVEF2_CA	LVEF2_CA	PVR	Valve_Typ	RIGHT_AR	PPM	Period
1.50	3	3	0	0	0		0	0	3
2.10	3	3	0	0	1	METALIC	0	0	3
1.10	#NULL!	#NULL!	1	1	1	METALIC	0	1	3
1.47	2	2	1	1	0		0	0	3
1.08	3	3	1	0	0		0	0	3
1.40	1	1	1	1	1	BIO	0	0	3
#NULL!	2	2	1	0	0		0	0	3
1.15	3	3	1	0	1	BIO	0	0	3
2.20	#NULL!	#NULL!	0	0	0		1	0	3
1.67	3	3	1	0	0		0	0	3
0.71	3	3	1	1	1	METALIC	1	0	2
1.22	2	2	1	0	0		1	0	2
1.36	#NULL!	#NULL!	1	1	0		0	0	2
0.96	#NULL!	#NULL!	1	0	1	BIO	0	0	1
1.70	3	3	0	0	1	METALIC	0	0	2
1.41	3	3	1	1	0		0	0	3
1.23	3	3	1	1	1	METALIC	0	0	1
1.69	3	3	1	0	0		0	0	2
1.40	#NULL!	#NULL!	1	0	1	BIO	0	0	2
0.81	2	2	1	1	0		0	0	1
2.16	3	3	0	0	0		1	0	2
#NULL!	3	3	1	0	1	BIO	0	0	1
0.38	2	2	1	1	1	BIO	0	0	1
1.66	3	3	0	0	0		0	0	1
1.34	3	3	0	0	1	BIO	0	0	1
1.59	3	3	1	0	0		0	0	3
1.04	3	3	1	0	0		0	0	1
0.79	3	3	1	0	0		0	0	1
2.41	3	3	0	0	0		0	0	3
0.70	1	1	1	1	0		0	0	1
1.08	3	3	1	0	0		0	0	1
0.48	3	3	1	0	1	BIO	0	0	1
1.69	3	3	0	1	0		0	0	1
0.47	3	3	1	1	1	BIO	1	1	1
0.97	2	2	1	0	0		0	0	1
1.77	3	3	1	0	0		0	0	3
1.88	3	3	1	1	0		0	0	2
0.88	3	3	1	1	0		0	0	2
0.66	2	2	1	0	0		0	0	1
0.71	3	3	1	0	0		1	0	1
1.41	#NULL!	#NULL!	1	0	0		1	0	1
1.88	2	2	0	0	0		0	0	2
0.82	3	3	1	0	1	BIO	1	0	1
0.68	2	2	1	0	1	BIO	0	0	1
1.35	2	2	1	1	0		1	0	3
1.47	3	3	1	0	0		0	0	3

0.84	2	2	1	0	1	BIO	1	0	1
1.50	3	3	0	0	0		0	0	2
1.06	3	3	0	0	0		0	0	1
1.64	#NULL!	#NULL!	0	1	0		0	0	1
#NULL!	#NULL!	#NULL!	1	0	0		0	0	1
0.74	3	3	1	1	0		0	0	1
1.04	1	1	1	0	0		1	0	3
0.81	2	2	1	0	0		0	0	1
1.25	#NULL!	#NULL!	1	0	1	BIO	0	0	1
1.45	3	3	1	1	0		0	0	3

FollowUp	time_to_PVR
1	#NULL!
1	15.00
1	24.00
0	#NULL!
1	#NULL!
1	11.00
1	#NULL!
1	13.00
1	#NULL!
1	#NULL!
1	18.00
0	#NULL!
1	#NULL!
1	17.50
1	#NULL!
1	#NULL!
1	15.00
1	#NULL!
1	3.00
0	#NULL!
1	#NULL!
1	14.00
1	#NULL!
1	#NULL!
1	10.00
1	#NULL!
0	#NULL!
0	#NULL!
1	#NULL!
1	#NULL!
1	#NULL!
1	5.00
1	#NULL!
1	4.00
1	#NULL!
0	#NULL!
1	#NULL!
0	#NULL!
1	#NULL!
1	#NULL!
1	#NULL!
1	#NULL!
1	10.00
1	6.00
1	#NULL!
1	#NULL!

1	6.00
1	#NULL!
1	#NULL!
1	#NULL!
1	#NULL!
1	#NULL!
1	#NULL!
1	#NULL!
1	31.00
1	#NULL!
